# Identification of a chromatin regulator signature and potential prognostic ability for adrenocortical carcinoma

**DOI:** 10.3389/fgene.2022.948353

**Published:** 2022-08-26

**Authors:** Junwu Li, Yuanzhen Jia, Lin Tang, Ronggui Zhang, Yuanfeng Zhang

**Affiliations:** ^1^ Department of Urology, The Second Affiliated Hospital of Chongqing Medical University, Chongqing, China; ^2^ Department of Rheumatology and Immunology, The Second Affiliated Hospital of Chongqing Medical University, Chongqing, China; ^3^ Department of Urology, Chongqing Emergency Medical Center, Chongqing, China

**Keywords:** chromatin regulator, adrenocortical carcinoma, prognosis, diagnosis, treatment

## Abstract

**Objective:** Adrenocortical carcinoma (ACC) is a rare malignant tumor. Chromatin regulators (CRs) can drive epigenetic changes, which have been considered as one of the most vital hallmarks of tumors. This study aimed to explore the CR signature for ACC in order to clarify the molecular basis of ACC’s pathogenic mechanism and provide novel methods to diagnose and treat ACC clinically.

**Methods:** This study obtained transcriptome sequencing datasets of ACC patients and sequencing data on normal adrenal tissues in TCGA and GTEx databases, respectively. Meanwhile, prognostic genes were selected through Lasso and Cox regression analyses. Using the transcriptome sequencing datasets of ACC patients downloaded from the GEO database to finish validation, we performed Kaplan–Meier (KM) analysis for evaluating the differential survival between low- and high-risk groups. Then, this work constructed the risk model for predicting ACC prognosis. TIMER 2.0 was employed to assess the differences in immune infiltration between the two groups. Furthermore, this work adopted the R package “pRRophetic” for exploring and estimating the sensitivity of patients to different chemotherapeutic agents.

**Results:** A 5-CR model was established to predict ACC survival, and the CR signature was confirmed as a factor in order to independently predict ACC patient prognosis. In addition, a nomogram composed of the risk score and clinical T stage performed well in the prediction of patients’ prognosis. Differentially expressed CRs (DECRs) were mostly associated with the cell cycle, base excision repair, colon cancer, gene duplication, homologous recombination, and other signaling pathways for the high-risk group. As for the low-risk group, DECRs were mainly enriched in allograft rejection, drug metabolism of cytochrome P450, metabolism of xenogeneic organisms by cytochrome P450, retinol metabolism, and other signaling pathways. According to TIMER analysis, the immune infiltration degrees of endothelial cells, M2 macrophages, myeloid dendritic cells, CD4^+^ Th1 cells, NKT cells, and M0 macrophages showed significant statistical differences between the high- and low-risk groups, and high infiltration levels of M0 and M2 macrophages were more pronounced in higher T stage (T3 and T4), N stage (N1), and clinical stages (III and IV). In addition, high-risk cases exhibited higher sensitivity to etoposide and doxorubicin. Additionally, low-risk patients had significantly decreased expression of RRM1 compared with high-risk cases, suggesting the better effect of mitotane treatment.

**Conclusion:** This study identified the DECRs, which might be related to ACC genesis and progression. The pathways enriched by these DECRs were screened, and these DECRs were verified with excellent significance for estimating ACC survival. Drug sensitivity analysis also supported the current clinical treatment plan. Moreover, this study will provide reliable ideas and evidence for diagnosing and treating ACC in the clinic.

## Introduction

ACC represents an uncommon malignant cancer, which has an annual morbidity of around 1-2/1,000,000 people (Else et al., 2014). It is also a frequently seen primary adrenal gland cancer (Chandrasekar et al., 2019), accounting for 6.8% of primary adrenal tumors (Lam, 1992), and it ranks second place among endocrine organ cancers, only second to thyroid cancer (TC) (Abe and Lam, 2021). ACC displays a high malignancy grade, and the 5-year survival rate is only 10%–20% in accordance with the statistics (Libé, 2015). ACC can occur at any age, with two peaks in childhood and the age of 50–70 years, and is more common in women (Fassnacht et al., 2009). ACC has rapid development, strong invasiveness, and dismal survival. Many patients have developed local invasion or distant metastasis (DM) when they are diagnosed. Based on the reports, the 5-year survival rates of stage I-IV ACC are 82%, 58%, 55%, and 13%, respectively (Allolio and Fassnacht, 2006). Recent epidemiological studies have indicated that the incidence of ACC increases year by year over the past 40 years, without any improvement in patient survival (Aufforth and Nilubol, 2014).

Epigenetic alterations are considered a vital hallmark of cancer. They are driven *via* CRs, the integral regulatory elements in epigenetics (Lu et al., 2018). According to their roles in epigenetics, CRs are mainly divided into three categories, namely, DNA methylating agents, histone modifiers, and chromatin remodeling agents (Plass et al., 2013). CRs are closely associated with each other. Further research shows that abnormal CR levels are related to various biological processes, such as inflammation (Marazzi et al., 2018), apoptosis (Li et al., 2020a), autophagy (Chu et al., 2020), and proliferation (Chen et al., 2020). This indicates that CR dysregulation may possibly generate disease occurrence, such as cancer. In recent years, an increasing number of studies have been conducted to screen key prognostic genes for ACC by bioinformatics analysis. However, CRs, as a key point of epigenetics, have not received corresponding attention. Therefore, this study aimed to explore the CR signature in ACC and further examine their functions in ACC prognosis with the purpose of clarifying ACC molecular basis and offering novel methods to diagnose and treat ACC in the clinic.

## Methods and materials

### Data acquisition

The transcriptome sequencing dataset for 79 ACC cases was downloaded from TCGA database (https://portal.gdc.cancer.gov). As normal samples were not included in TCGA-ACC, UCSC Xena was applied to obtain sequencing data on 128 normal adrenal tissue samples from the GTEx database. Thereafter, the top 100 CR-encoding genes with the greatest impact on ACC patients were obtained from the Facer database (http://bio-bigdata.hrbmu.edu.cn/FACER/). As a validation cohort, we downloaded the GSE10927 dataset with transcriptome sequencing data on 33 ACC cases, 22 adrenocortical adenoma (ACA) cases, and 10 normal adrenal tissue samples from the GEO database (https://ncbi.nlm.nih.gov) in order to confirm the differential expression of CR-encoding genes. In addition, we also downloaded the transcriptome sequencing and prognosis data on 23 ACC patients from the GSE33371 dataset to verify the reliability of the prognosis prediction model.

### Differential analysis

All data were corrected to the log2 (FPKM+0.001) format for further comparison. Meanwhile, “Limma” in the R package was adopted for correcting the offset of datasets and performing differential analysis. The absolute value of logFC greater than 1 and *p* < 0.05 were applied as the thresholds to select differentially expressed genes (DEGs). Afterward, up-and downregulated genes were, respectively, explored, and the DECRs in ACC were obtained after intersecting with CR-encoding genes.

### Construction of the prognosis prediction model

Univariate Cox regression was conducted to analyze DECRs’ effect on prognosis, and the significant prognostic genes (*p* < 0.05) screened were later incorporated into Lasso regression analysis, followed by the construction of the prognosis prediction model. Thereafter, based on the median risk score, patients were classified into a low- or high-risk group. Subsequently, receiver operating characteristic (ROC) curves were plotted to assess whether the prognostic model was of high prognostic power. Afterward, univariate and multivariate COX regression analyses were conducted for assessing the effect of risk scores on ACC survival. In addition, we also utilized the R package “rms” for drawing the nomogram of risk scores for ACC patients and the 1-, 3-, and 5-year calibration curves. The model C-index was also calculated, and the effect of DECRs on overall survival (OS) was assessed by adopting Kaplan–Meier (KM) survival analysis.

### Functional enrichment analysis

The enrichment of DECRs in Gene Ontology_biological process (GO_BP), cellular component (GO_CC), and molecular function (GO_MF) pathways was assessed using the R package “enrichplot,” respectively. Furthermore, GSEA software was employed to explore the significantly different GO_BP, GO_CC, GO_MF, and Kyoto Encyclopedia of Genes and Genomes (KEGG) pathways (FDR < 0.25) between low- and high-risk patients.

### Immune functional analysis

The infiltration levels of immune cells within TCGA-ACC cancer tissues under seven algorithms were obtained from TIMER 2.0. The differences between low- and high-risk patients were evaluated.

### Drug sensitivity analysis

Mitotane is currently the most common and effective agent used for adjuvant therapy after ACC surgery and metastatic ACC. The expression of RRM1 in the tumor is a good predictor of the efficacy of mitotane therapy, and its low expression usually indicates the response to mitotane therapy. Therefore, the expression levels of RRM1 in ACC patients were extracted in order to compare the mitotane response in high- and low-risk patients. In addition, etoposide, doxorubicin, and cisplatin are also the commonly used chemotherapeutic agents for metastatic ACC. As a result, “pRRophetic” of the R package was utilized for predicting chemotherapeutic sensitivity based on the whole-transcriptome information of patients.

## Results

### Establishment of a chromatin regulator signature

**FIGURE 1:**
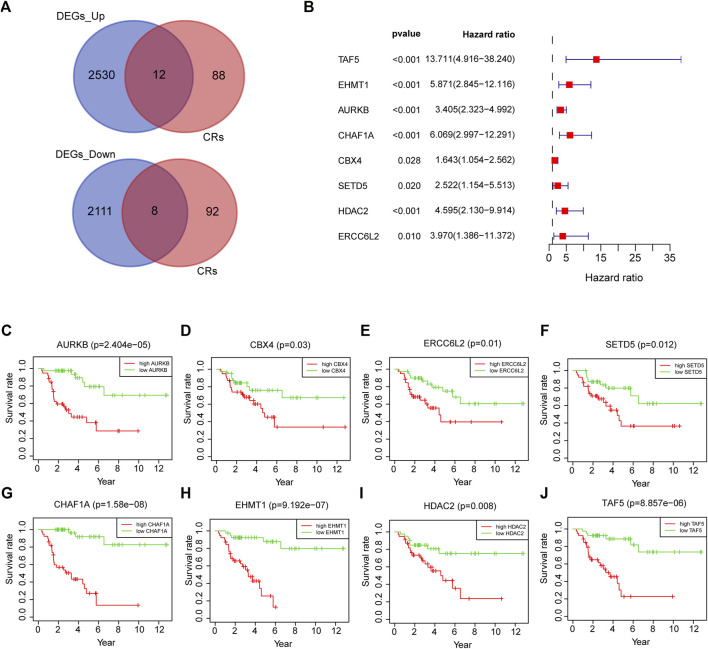
20 DECRs selected from the intersection of DEGs and CRs **(A)**. Univariate Cox regression of eight DECRs that showed a prognostic value **(B)**. KM survival analysis of eight DECRs that showed a prognostic value **(C–J)**.

**FIGURE 2:**
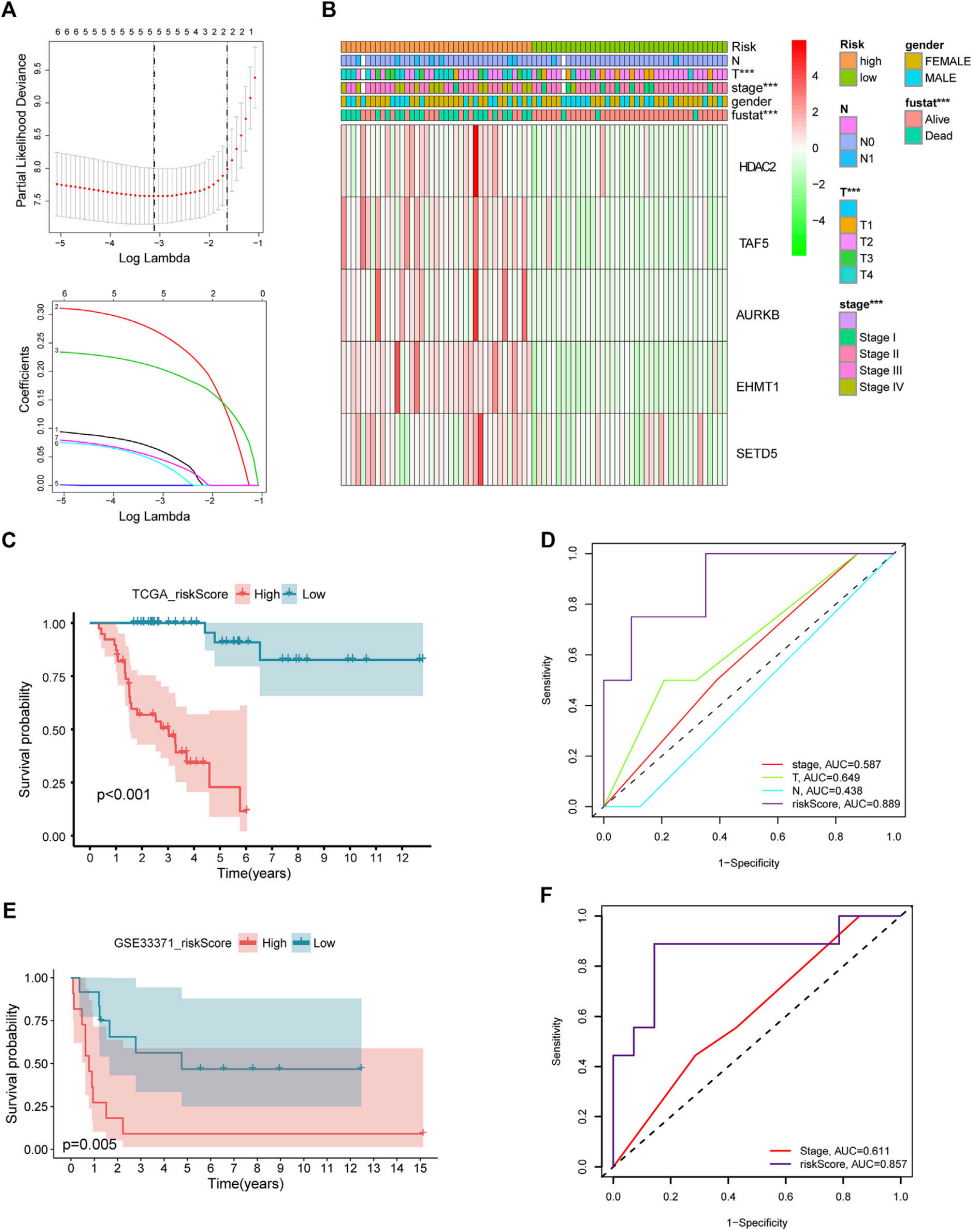
Lasso Cox regression of DECRs **(A)**. Expression of five selected DECRs in different clinical features groups **(B)**. KM survival analysis of low- and high-risk patients in test cohort **(C)**. ROC analysis of clinical stage, clinical T stage, clinical N stage and riskscore in test cohort **(D)**. KM survival analysis of low- and high-risk patients in validation cohort **(E)**. ROC analysis of stage and riskscore in validation cohort **(F)**.

After intersecting TCGA-ACC dataset with the ACC CR dataset, a total of 20 DECRs were screened, among which 12 showed downregulation whereas 8 exhibited upregulation (Figure 1A). According to the abovementioned dysregulated CRs, univariate Cox regression was adopted for exploring their prognostic significance. As a result, only 8 out of these 20 DECRs showed a prognostic value (Figures 1B–J). Later, this work utilized Lasso Cox regression for constructing the prognosis prediction characteristics for ACC patients. The risk model based on five genes (*TAF5*, *EMHT1*, *AURKB*, *SETD5*, and *HDAC2*) was successfully constructed (Figure 2A). For verification, we employed the expression data extracted from the GSE10927 dataset of the GEO database to intersect with the ACC CR dataset with the aim of performing expression differences. The result proved that these CRs have significant expression differences between ACC and other tissues including ACA and normal adrenal tissues (Supplementary Figure S1). Then, we determined the risk score by correlation coefficients of the 5 DECRs: Risk score = (0.0637 × TAF5 level) + (0.2699 × EMHT1 level) + (0.2068 × AURKB level) + (0.0418 × SETD5 level) + (0.0482 × HDAC2 level) (Table 1). Finally, ACC cases were classified into two (low- or high-risk) groups, in accordance with the median risk score. As a result, high-risk patients showed an obviously increased death proportion compared with low-risk counterparts (*p* < 0.001), suggesting the negative correlation of the risk score with patient survival (Figures 2B,C). Based on ROC analysis, the CR signature achieved a 0.889 prognostic accuracy in TCGA dataset (Figure 2D). The results of the validation cohort also proved the significant difference between high- and low-risk groups (*p* < 0.05), and the ROC analysis indicated a 0.857 prognostic accuracy of the CR signature (Figures 2E,F).

**TABLE 1:** Correlation coeffcients of five selected DECRs.

Gene	Coef
*TAF5*	0.063716491
*EHMT1*	0.269857731
*AURKB*	0.206839715
*SETD5*	0.041749529
*HDAC2*	0.048168878

### Independent prognostic indicators of chromatin regulator signature

Univariate and multivariate Cox regression analyses were conducted to demonstrate the feasibility of the CR signature in order to independently predict prognosis. According to the results of univariate regression, the clinical stage, clinical T stage, and risk score showed significant relation to ACC survival (*p* < 0.001). Upon multivariate regression, the clinical T stage and risk score remained significantly associated with ACC survival (*p* < 0.05) (Table 2). In the validation cohort, univariate Cox regression also showed that the risk score was notably associated with ACC survival (*p* < 0.05) (Table 3). All the aforementioned results indicated that the CR signature was the independent prognostic indicator for ACC patients.

**TABLE 2:** Univariate and multivariate regression of CR signature and other clinical features in test cohort.

Characteristic	Univariate analysis		Multivariate analysis	
	Hazard ratio (95% CI)	*p*-value	Hazard ratio (95% CI)	*p*-value
Gender	1.056 (0.490–2.276)	0.890	—	—
Stage	2.903 (1.844–4.569)	<0.001	1.198 (0.481–2.983)	0.699
T	3.364 (2.098–5.393)	<0.001	3.222 (1.302–7.971)	0.011
N	2.058 (0.774–5.472)	0.148	—	—
Risk score	1.006 (1.004–1.008)	<0.001	1.005 (1.003–1.008)	<0.001

**TABLE 3:** Univariate and multivariate regression of CR signature and other clinical features in validation cohort.

Characteristic	Univariate analysis		Multivariate analysis	
	Hazard ratio (95% CI)	*p*-value	Hazard ratio (95% CI)	*p*-value
Gender	1.358 (0.467–3.954)	0.574	—	—
Stage	1.700 (1.036–2.790)	0.036	2.589 (1.353–4.954)	0.004
Risk score	12,191.428 (18.539–8,017,342.094)	0.004	1676261.622 (370.856–7576636516.957)	<0.001

### Relationship of chromatin regulator signature with clinical features

**FIGURE 3:**
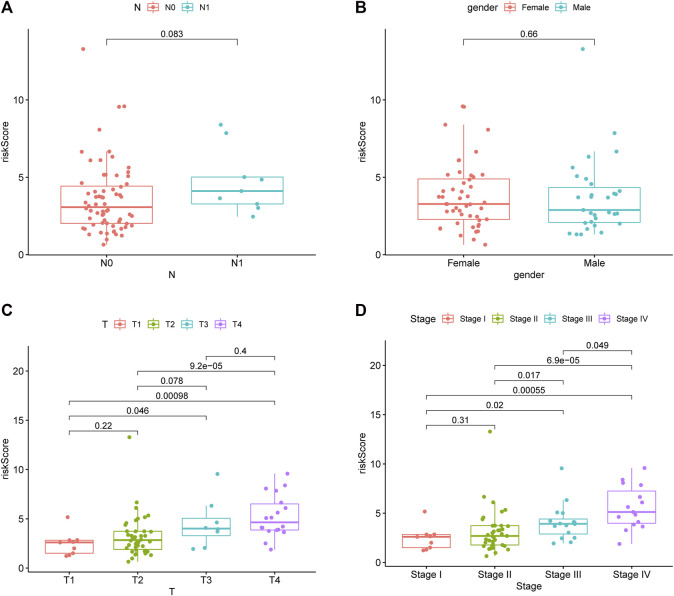
Riskscore in different gender, clinical stage, clinical T stage and clinical N stage.

This study utilized a chi-squared test to explore the involvement of CR prognostic features in ACC occurrence and progression. As a result, the clinical T stage (*p* < 0.001) and clinical stage (*p* < 0.001) were significantly different between high- and low-risk groups, while no difference was detected in gender or clinical N stage (*p* > 0.05) (Figures 3A,B). In addition, further subgroup analyses were performed to investigate whether the CR signature was significant for prognosis prediction. According to the obtained results, the CR signature exhibited excellent performance in predicting I−III (*p* = 0.02), I−IV (*p* < 0.001), II−III (*p* = 0.017), II−IV (*p* < 0.001), T1−T3 (*p* = 0.046), T1−T4 (*p* < 0.001), and T2−T4 (*p* < 0.001) stages, while the CR signature performed poorly concerning its prognosis prediction performance at T1−T2 and I−II stages (*p* > 0.05) (Figures 3C,D).

### Construction and verification of the nomogram

**FIGURE 4:**
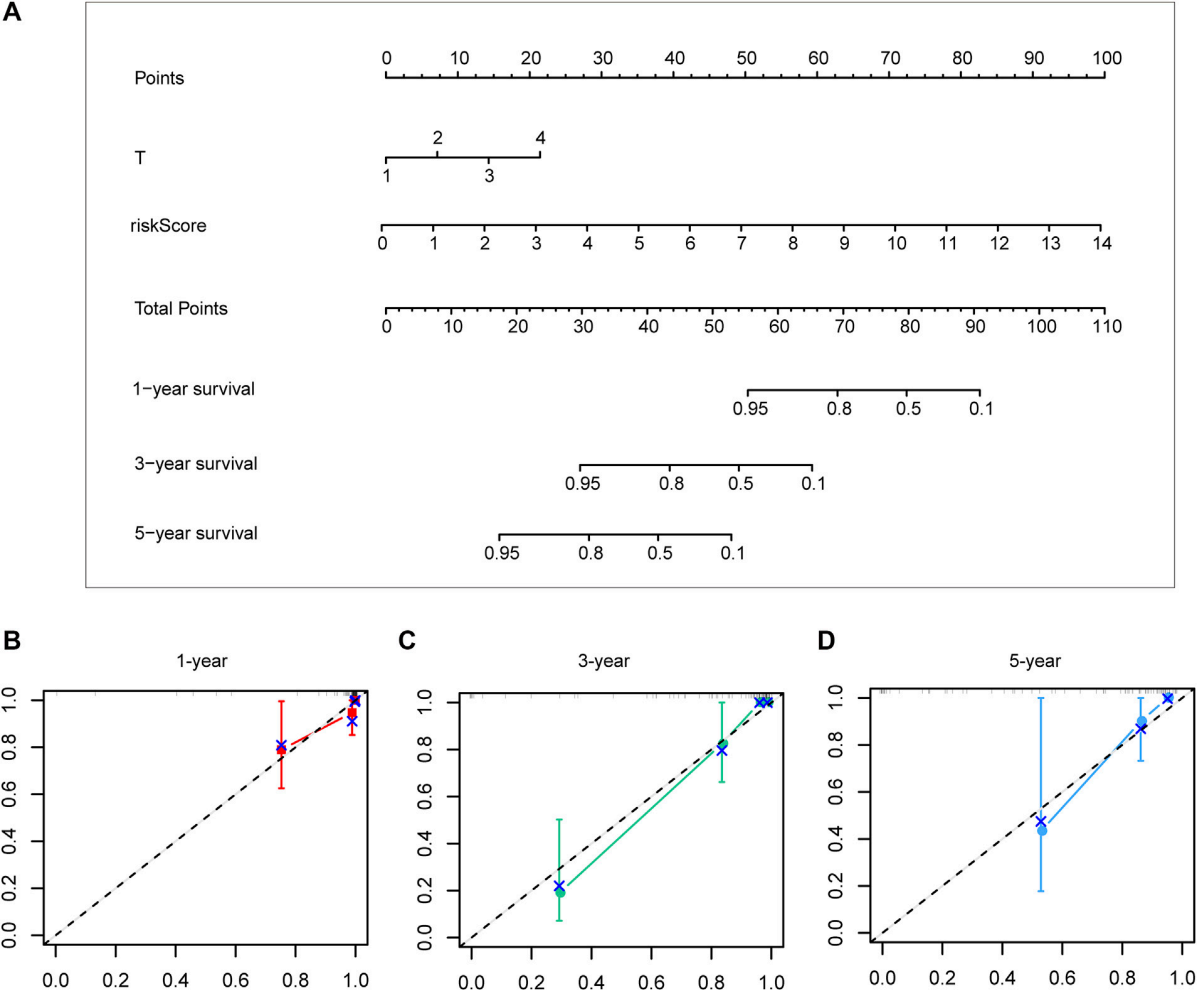
A nomogram constructed by clinical T stage and riskscore in test cohort **(A)**. Calibration curve predicting 1-, 3-, and 5-year patient prognosis **(B–D)**.

**FIGURE 5:**
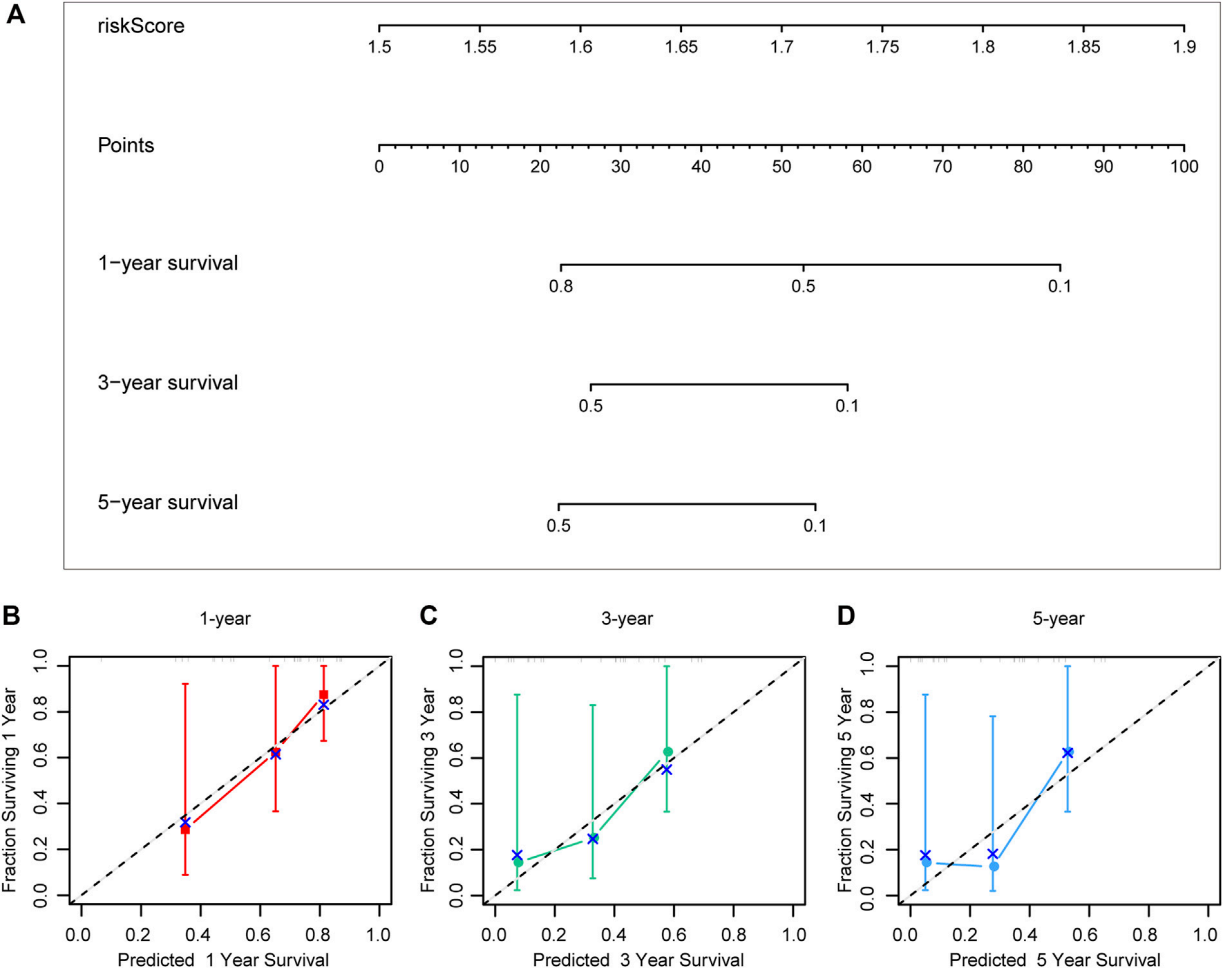
A nomogram constructed by riskscore in validation cohort **(A)**. Calibration curve predicting 1-, 3-, and 5-year patient prognosis **(B–D)**.

Different prognostic indicators were incorporated into the nomogram to graphically assess survival probabilities for different patients in the preoperative stage. The nomogram incorporating the clinical T stage and risk score was constructed to better predict 1-, 3-, and 5-year patient prognosis (Figure 4A). Based on the calibration curve, there was good consistency between the measured patient survival and the estimated survival (Figures 4B–D). In addition, the nomogram achieved a C-index of 0.929, proving its good predictive power. During validation, due to missing the clinical T stage date of patients, we constructed a nomogram based only on the risk score and used it to predict 1-, 3-, and 5-year patient prognosis in the validation cohort. The calibration curve also showed good consistency between the measured patient survival and the estimated survival. At the same time, the C-index of the nomogram in the validation cohort was 0.726 (Figures 5A–D).

### Functional annotation and gene set enrichment analyses

**FIGURE 6:**
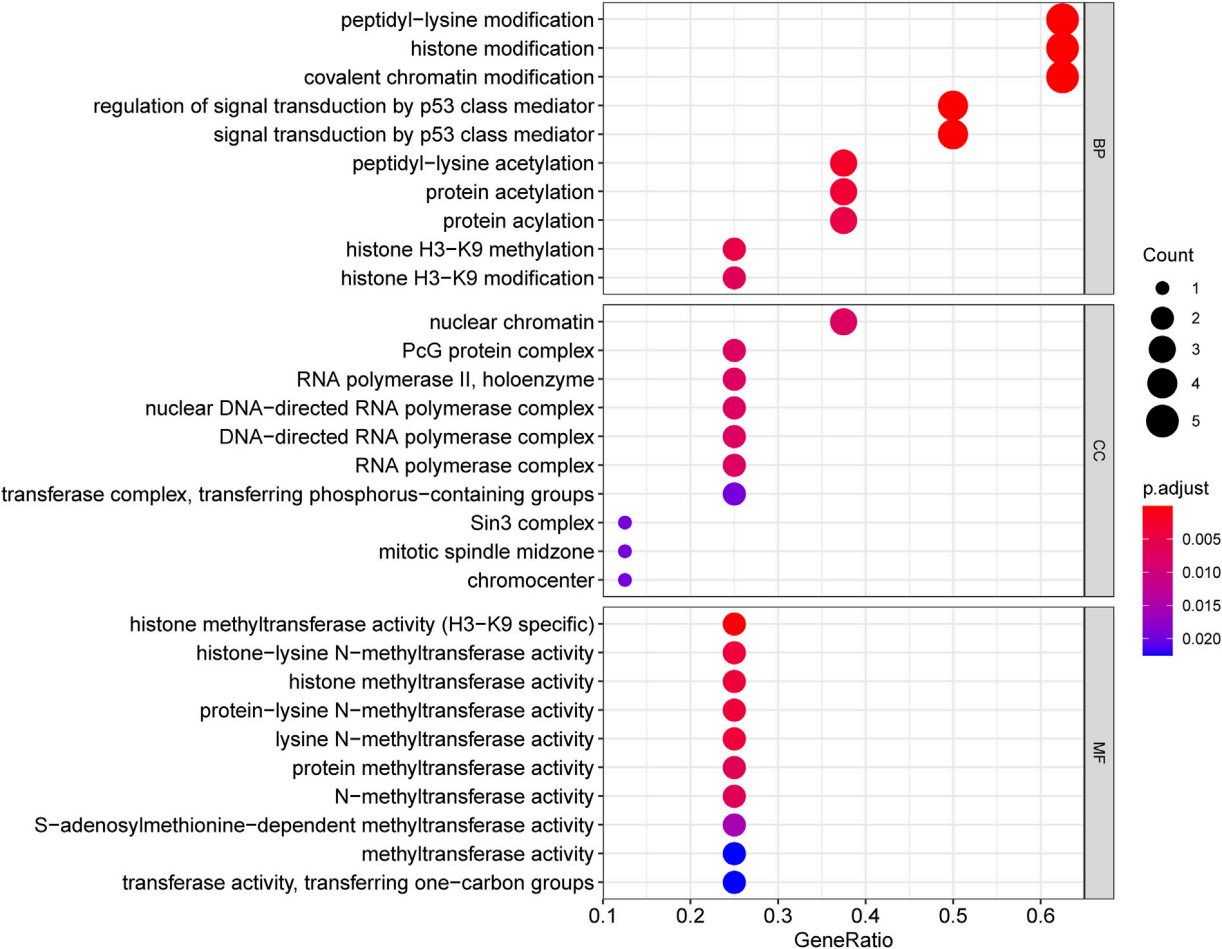
GO_BP, CC and MF analysis of 20 DECRs.

**FIGURE 7:**
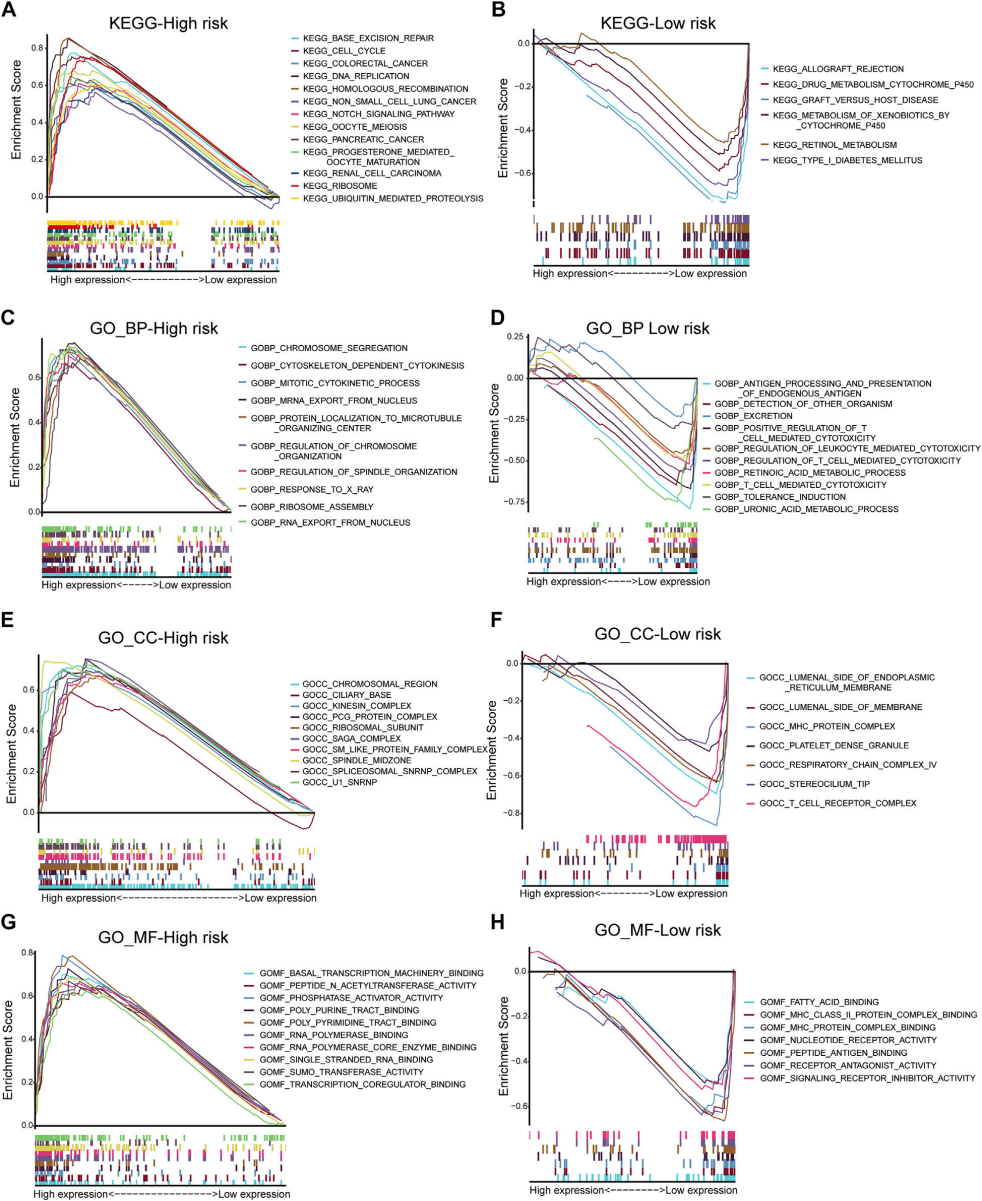
KEGG analysis of low- and high-risk groups **(A,B)**. GSEA analysis of BP, CC and MF in low- and high-risk groups **(C–H)**.

This study performed GO and KEGG analyses to explore possible functions of DECRs. According to BP analysis results, these 20 DECRs were significantly related to histone modification, peptidyl-lysine modification, and covalent chromatin modification. Based on the analysis of CC, these 20 DECRs were mainly associated with PcG protein complexes, nuclear chromatin, and RNA polymerase II. MF analysis demonstrated that the 20 DECRs were mainly enriched in the histone methyltransferase activity, histone–lysine N-methyltransferase activity, and protein–lysine N-methyltransferase activity (Figure 6). Furthermore, KEGG analysis showed that the signaling pathways including base excision repair, cell cycle, colon cancer, gene duplication, and homologous recombination were enriched in the high-risk group. Meanwhile, the low-risk group was associated with signaling pathways such as allograft rejection, drug metabolism of cytochrome P450, metabolism of xenogeneic organisms by cytochrome P450, and retinol metabolism (Figures 7A,B). At the same time, to better clarify the molecular basis of CR signature, gene set enrichment analysis (GSEA) was conducted. As a result, high-risk patients were associated with chromosome segregation, chromosome region, transcriptional binding, and other pathways, whereas low-risk patients were mostly associated with antigen processing and internalization, originated antigen presentation, luminal side of the endoplasmic reticulum, fatty acid binding, and other pathways (Figures 7C–H).

### Immune infiltration analysis of chromatin regulator signature

**FIGURE 8:**
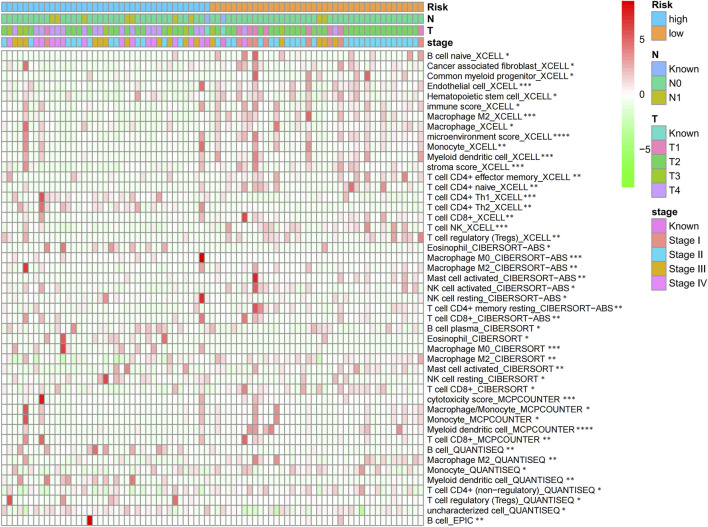
Heat map of CR signature with immune infiltration by TIMER analysis.

According to the TIMER analysis, relations of the CR signature with immune infiltration were shown by a heat map (Figure 8). As a result, the immune infiltration degrees of endothelial cells, M2 macrophages, myeloid dendritic cells, CD4^+^ Th1 cells, NKT cells, and M0 macrophages exhibited significant statistical differences between the high- and low-risk groups, and high infiltration levels of M0 and M2 macrophages were more pronounced in a higher T stage (T3 and T4), N stage (N1), and clinical stages (III and IV).

### Drug sensitivity test

**FIGURE 9:**
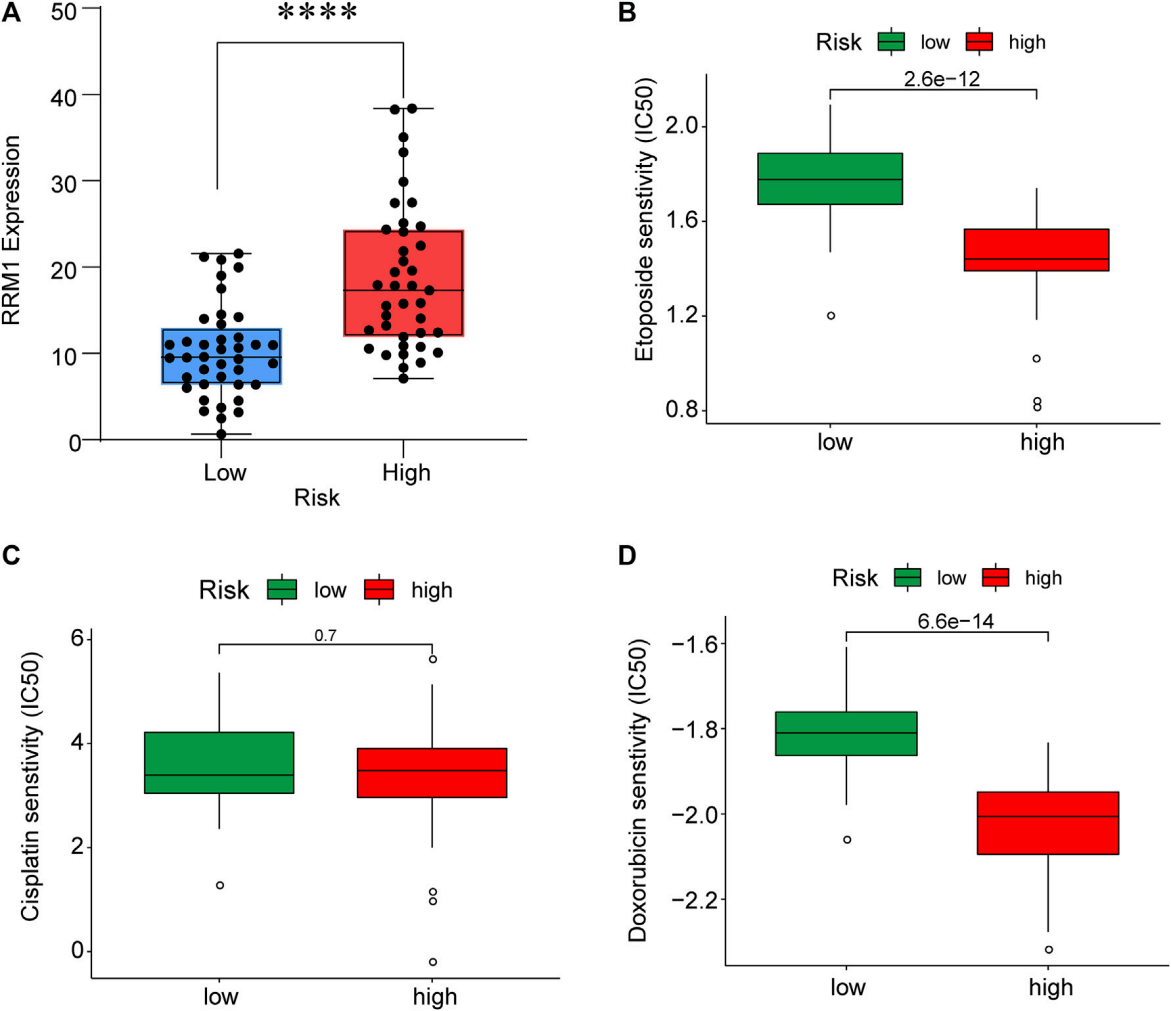
RRM1 expression in low- and high-risk groups **(A)**. Etoposide, cisplatin and doxorubicin senstivity in low- and high-risk groups **(B–D)**.

To improve the therapeutic efficacy in ACC cases, this study explored the difference in common chemotherapeutic agent sensitivity in ACC. Based on the results of the GDSC database analysis, for high-risk patients, their IC50 values of etoposide and doxorubicin increased compared with low-risk patients, suggesting the higher sensitivity of high-risk patients to these drugs (Figure 9). Meanwhile, RRM1 levels were significantly elevated among high-risk patients compared with low-risk patients (*p* < 0.001), which indicated the better curative effect of mitotane on low-risk cases.

## Discussion

Previous studies have shown that most ACC is sporadic of unknown origin, while a minority can be attributed to some hereditary neoplastic syndromes, including Li-Fraumeni syndrome, Lynch syndrome, MEN-1, and familial adenomatous polyposis (Vaidia et al., 2019). According to whether the tumor has an endocrine function, ACC is categorized into functional and non-functional types. Functional ACC can be diagnosed easier, and it clinically manifests as hypercortisolism, Cushing’s syndrome, and primary hyperaldosteronism, while non-functional ACC often manifests as nonspecific tumor-induced symptoms due to its insidious onset. Because ACC does not show any obvious early onset characteristics, 70% of such cases are already in stages III-IV when they are diagnosed (Bharwani et al., 2011; Fay et al., 2014). Currently, the treatment methods for ACC include surgery, chemotherapy, and radiotherapy, but none of them can achieve ideal therapeutic effects (Allolio and Fassnacht, 2006). The discovery of novel predictive factors for the diagnosis and prognosis of ACC will help clinicians assess the risk for patients and formulate the targeted treatment strategies. With the development of information technology, study on the diagnostic and prognostic markers for ACC has gradually emerged in recent years. For instance, He ZJ screened 15 key genes (*CXCR6*, *SELL*, *P2RY13*, *GNG8*, *OMD*, *ABI3BP*, *OGN*, *FBLN1*, *LOXL1*, *ELN*, *CTSK*, *HGF*, *SH3GL3*, *F13A1*, and *GTPBP2*) based on the mRNA-seq sequencing data and the stem cell index established according to TCGA-ACC mRNA profiles. In addition, they also pointed out that GTPBP2 was the only key gene with prognostic significance (He, 2021). After that, Hu DF et al*.* employed the GEO database to screen the differential core genes that were upregulated (*RACGAP1*, *CCNB1*, *TYMS*, *MAD2L1*, *NCAPG*, and *CDK1*) and downregulated (*IGF1*, *CXCL12*, *TLR4*, *TGFBR2*, and *HGF*). However, the authors did not investigate the value of these genes. Qi et al. (2021) and Zhou et al. (2022) analyzed more updated GEO database samples on the aforementioned basis. As a result, *CCNB1*, *CCNA2*, *CDK1*, *BUB1B*, *MAD2L1*, *RRM2*, *TPX2*, *AURKA*, *TOP2A*, *ZWINT*, and *NCAPG* were found to be closely related to prognosis. Nevertheless, further exploration of their value was lacking. In other words, the data-supported reliable clinical diagnostic and prognostic indicators are still needed for ACC. Recently, CRs have been increasingly suggested to make different effects on carcinogenesis, while little existing research has systemically examined CRs and explored their clinical value for ACC.

Based on the aforementioned starting point, this study selected a total of eight DECRs with upregulation and 12 DECRs with downregulation. According to univariate Cox regression, eight of them showed a prognostic value, and five genes were successfully constructed by adopting the Lasso Cox model. Subsequently, we verified the expression differences of the aforementioned genes by using the GEO database. The result demonstrated that *TAF5*, *EMHT1*, *AURKB*, and *SETD5* all showed significant expression differences in line with the results except *HDAC2*. After discussion, we believed that the difference in *HDAC2* may be caused by the small sample size. Then, correlation coefficients of five DECRs were determined to calculate the risk score as follows (0.0637 × TAF5 expression) + (0.2699 × EMHT1 expression) + (0.2068 × AURKB expression) + (0.0418 × SETD5 expression) + (0.0482 × HDAC2 expression). According to the median risk score, ACC cases were classified into a low- or high-risk group. As a result, high-risk patients showed significantly increased deaths compared with low-risk counterparts (*p* < 0.001), suggesting a negative correlation between the risk score and prognosis. Based on ROC analysis, the CR signature achieved a prognostic accuracy of 0.889. The results of the validation cohort were consistent with this finding, and the ROC analysis showed the prognostic accuracy of the CR signature was 0.857 in the validation cohort, which reflected the superior prognostic value of the CR signature. Afterward, univariate and multivariate Cox regression analyses were conducted. As a result, the CR signature independently predicted ACC prognosis. According to the chi-squared test, the clinical T stage (*p* < 0.001) and clinical stage (*p* < 0.001) were significantly different between the two groups, while age and clinical N stage did not exhibit any difference (*p* > 0.05). Furthermore, our study proved that the CR signature exhibited excellent performance in predicting I−III (*p* = 0.02), I−IV (*p* < 0.001), II−III (*p* = 0.017), II−IV (*p* < 0.017) T1−T3 (*p* = 0.046), T1−T4 (*p* < 0.001), and T2−T4 (*p* < 0.001) stages. Finally, the nomogram incorporating the clinical T stage and risk score was constructed. According to the calibration curve, the measured patient survival showed high consistency with the estimated one. Our nomogram achieved a C-index of 0.929, confirming its good prediction performance. Because one of the main purposes of our research was to explore the prognostic ability of the CR signature, although the clinical T stage data on patients in the validation cohort were missing, we still constructed a nomogram based on the risk score for validation. The results also showed good consistency between the measured patient survival and estimated survival. At the same time, the C-index of the nomogram in the validation cohort was 0.726. It is of note that previous studies have revealed that surgical methods, surgical margins, pathological features, and Ki-67 proliferation index are also associated with poor prognosis in ACC. Nevertheless, databases including TCGA and GEO cannot provide detailed data on the corresponding aspects of patients. This study concentrated on building a preoperative, less traumatic predictive risk model. Therefore, only the clinical T stage and risk score were included to build the nomogram. In addition, the results also confirmed the excellent predictive ability of the model.

As the 100-kDa subunit of the universal transcription factor TFIID, human TAF5 makes a vital effect on assembling the 1.2-MDa TFIID complex. In a study on human papillomavirus (HPV), in the context of oral squamous cell carcinoma (OSCC), *TAF5* and other genes showed high enrichment into HPV-positive somatic mutations, which mostly influence the HPV oncoprotein-targeted host pathways including pRB and p53 pathways. They also play important roles in disrupting the host’s defense against viral infection and are potentially involved in nuclear factor-kappa B (NF-kB) and interferon (IFN) signaling (Gillison et al., 2019). Lee J and colleagues explored the effect of EHMT1 on lung cancer. According to the obtained results, EHMT1 was significantly related to apoptosis and the cell cycle process and had an important impact on regulating the apoptosis and cell cycle of tumor cells by regulating the expression of *CDKN1A* (Lee et al., 2021). Watson et al. (2019) also confirmed in their study on high-grade serous ovarian cancer (HGCOC) that disruption of EHMT1/2 sensitized HGSOC cells to PARP inhibitors (PARPi). In addition, the authors also proposed a potential mechanism through DNA damage and cell cycle dysregulation (Watson et al., 2019). As a pan-cancer marker, AURKB is related to different tumor occurrences and development, including hepatocellular carcinoma (HCC) (Yang et al., 2022), bladder cancer (BLCA) (Tang and Wang, 2019), breast cancer (BRCA) (Zhang et al., 2021), lung adenocarcinoma (LUAD) (Ding et al., 2019), and osteosarcoma (Shan, 2021), exhibiting certain prognostic significance. Wang et al. (2020) identified SETD5 as a major driver of resistance to MEK1/2 (MEKi) in pancreatic ductal adenocarcinoma (PDAC), revealing that SETD5 was a key mediator of acquired resistance to MEKi therapy in PDAC. In addition, Chen et al. (2021) also confirmed that SETD5 promoted the cancer stem cell properties of non-small cell lung cancer (NSCLC) by attenuating the PI3K/Akt/mTOR pathway activation. Currently, the abnormal expression of *HDAC2* in different cancers has been widely confirmed, which is associated with cancer proliferation, invasion, migration, and drug resistance. HDAC2 also participates in tumor metabolism and influences the clinical diagnosis, treatment, and prognosis of cancers. In tumor cells, *HDAC2* acts as both a tumor-promoting gene and a tumor suppressor gene. In addition, its specific role is related to its target genes and pathways involved in various malignant tumors.

This study combined these five key CR genes at the ACC level for the first time and verified their unique prognostic and diagnostic significance. Based on GO and KEGG analyses, the BP analysis revealed the significant involvement of 20 DECRs in covalent chromatin modification, peptidyl-lysine modification, and histone modification. Analysis of CC revealed the significant enrichment of 20 DECRs in nuclear chromatin, PcG protein complexes, and RNA polymerase II. MF analysis showed that the 20 DECRs were mainly located in the histone methyltransferase activity, histone-lysine N-methyltransferase activity, and protein–lysine N-methyltransferase activity. Moreover, KEGG analysis showed that the signaling pathways including the base excision repair, cell cycle, colon cancer, gene duplication, and homologous recombination were enriched in the high-risk group. Apart from that, signaling pathways such as allograft rejection, drug metabolism of cytochrome P450, metabolism of xenogeneic organisms by cytochrome P450, retinol metabolism, and other signaling pathways were mostly related to low-risk patients. Based on GSEA results, the high-risk group was mainly associated with chromosome segregation, chromosome region, transcriptional binding, and other pathways, whereas low-risk groups were mostly related to antigen processing and presentation of endogenous antigens, luminal side of the endoplasmic reticulum, and fatty acid binding. Considering that ACC is a type of malignant endocrine tumor, this study also attempted to explore the relationship between these five DECRs and endocrine function. It is interesting to find that these five CRs are rarely discovered to be involved in some key endocrine metabolic pathways in previous studies. Among the enriched pathways, only cytochrome P450-related pathways have been shown to regulate aldosterone biosynthesis and participate in the pathogenesis of primary hyperaldosteronism (Bassett et al., 2004; Zennaro et al., 2012). According to other pathway enrichment results, it could be speculated that CRs more probably promote the occurrence and development of ACC by influencing cell division, cell cycle, and other links, rather than changing the level of hormone metabolism.

Through TIMER analysis, this study proved that the immune infiltration degrees of endothelial cells, M2 macrophages, myeloid dendritic cells, CD4^+^ Th1 cells, NKT cells, and M0 macrophages exhibited significant statistical differences between the high- and low-risk groups, and high infiltration levels of M0 and M2 macrophages were more pronounced in the higher T stage (T3, T4), N stage (N1), and clinical stages (III, IV). Macrophages, also known as tumor-associated macrophages (TAMs), block tumor immunity by producing immunosuppressive molecules and inducing immune tolerance, thereby generating a tumor microenvironment (TME) favorable for immune heterogeneity. Studies have proved that TAMs are involved in various biological events, including epithelial-mesenchymal transition, immune escape, tumor angiogenesis, and cancer metastasis (Li et al., 2020b), which are also likely to be the main mechanisms that these CRs affect the poor prognosis of ACC in terms of immune infiltration. According to our sensitivity difference analysis of common chemotherapeutic agents in ACC, for high-risk patients, their IC50 values of etoposide and doxorubicin increased compared with those of low-risk patients, suggesting the higher drug sensitivity of high-risk patients. Mitotane is currently the most commonly used and effective oral drug for the treatment of ACC (Author Anonymous, 2021). The low expression of the *RRM1* gene has been confirmed to be related to mitotane efficacy (Tang et al., 2020). Therefore, this study investigated *RRM1* expression based on the aforementioned analyses. As a result, high-risk patients had markedly increased RRM1 expression relative to low-risk counterparts (*p* < 0.001), indicating that mitotane had a better therapeutic efficacy in low-risk cases. Certainly, certain limitations should be noted in this work. For example, the mechanism by which these CRs regulate ACC cell biology should be further verified through further experiments. In addition, more multi-center clinical trials are also needed for verifying that our prognosis prediction model is practicable. There are still some challenges to be encountered, given the clinical rarity of ACC.

## Conclusion

To conclude, this study identified the DECRs that were possibly related to ACC genesis and progression, screened pathways enriched by these DECRs, and verified the excellent value of these DECRs in prognosis prediction for ACC cases. Moreover, the drug sensitivity of DECRs was also analyzed. Although more investigations are warranted for verifying our conclusions, this study provides reliable ideas and evidence for the clinical diagnosis and treatment of ACC.

## Data Availability

The original contributions presented in the study are included in the article/Supplementary Materials; further inquiries can be directed to the corresponding author.

## Glossary

ACC: adrenocortical carcinoma
ACA: adrenocortical adenoma
CRs: chromatin regulators
KM: Kaplan–Meier analysis
MPCs: myeloid progenitor cells
NK: natural killer cells
TC: thyroid cancer
DM: distant metastasis
DEGs: differentially expressed genes
DECRs: differentially expressed CRs
ROC: receiver operating characteristic curves
OS: overall survival
GO_BP: Gene Ontology_biological process
GO_CC: Gene Ontology_cellular component
GO_MF: Gene Ontology_molecular function
KEGG: Kyoto Encyclopedia of Genes and Genomes pathways
HPV: human papillomavirus
OSCC: oral squamous cell carcinoma
NF-kB: Nuclear factor-kappa B
IFN: interferon signaling
HGCOC: high-grade serous ovarian cancer
PARPi: PARP inhibitors
HCC: hepatocellular carcinoma
BLCA: bladder cancer
BRCA: breast cancer
LUAD: lung adenocarcinoma
PDAC: pancreatic ductal adenocarcinoma
NSCLC: non-small cell lung cancer
TAM: tumor-associated macrophage
TME: tumor microenvironment

## References

[B1] Abe I. Lam A. K. (2021). Anaplastic thyroid carcinoma: Updates on WHO classification, clinicopathological features and staging. Histol. Histopathol. 36 (3), 239–248. 10.14670/HH-18-277 33170501

[B2] Allolio B. Fassnacht M. (2006). Clinical review: Adrenocortical carcinoma: clinical update. J. Clin. Endocrinol. Metab. 91 (6), 2027–2037. 10.1210/jc.2005-2639 16551738

[B3] Aufforth R. D. Nilubol N. (2014). Emerging therapy for adrenocortical carcinoma. Int. J. Endocr. Oncol. 1 (2), 173–182. 10.2217/ije.14.13 25635221 PMC4307842

[B5] Bassett M. H. White P. C. Rainey W. E. (2004). The regulation of aldosterone synthase expression. Mol. Cell. Endocrinol. 217, 67–74. 10.1016/j.mce.2003.10.011 15134803

[B6] Bharwani N. Rockall A. G. Sahdev A. Gueorguiev M. Drake W. Grossman A. B. (2011). Adrenocortical carcinoma: the range of appearances on CT and MRI. AJR. Am. J. Roentgenol. 196 (6), W706–W714. 10.2214/AJR.10.5540 21606258

[B7] Chandrasekar T. Goldberg H. Klaassen Z. Wallis C. J. D. Woon D. T. S. Herrera-Caceres J. O. (2019). The who, when, and why of primary adrenal malignancies: Insights into the epidemiology of a rare clinical entity. Cancer 125 (7), 1050–1059. 10.1002/cncr.31916 30561782

[B8] Chen J. Wang F. Xu H. Xu L. Chen D. Wang J. (2020). Long non-coding RNA SNHG1 regulates the wnt/β-catenin and PI3K/AKT/mTOR signaling pathways via EZH2 to affect the proliferation, apoptosis, and autophagy of prostate cancer cell. Front. Oncol. 10, 552907. 10.3389/fonc.2020.552907 33194612 PMC7643000

[B9] Chen Q. Sun Z. Li J. Zhang D. Guo B. Zhang T. (2021). SET domain-containing protein 5 enhances the cell stemness of non-small cell lung cancer via the PI3K/Akt/mTOR pathway. J. Environ. Pathol. Toxicol. Oncol. 40 (2), 55–63. 10.1615/JEnvironPatholToxicolOncol.2021036991 33822517

[B10] Chu Y. Chen W. Peng W. Liu Y. Xu L. Zuo J. (2020). Amnion-derived mesenchymal stem cell exosomes-mediated autophagy promotes the survival of trophoblasts under hypoxia through mTOR pathway by the downregulation of EZH2. Front. Cell Dev. Biol. 8, 545852. 10.3389/fcell.2020.545852 33304896 PMC7693549

[B11] Ding H. Ye Y. An H. Gao Q. Zhong Y. (2019). Expression of key genes in lung adenocarcinoma and its prognostic significance. Chin. J. Comp. Med. 29 (10), 54–60. 10.3969/j.issn.1671-7856.2019.10.010

[B12] Else T. Kim A. C. Sabolch A. Raymond V. M. Kandathil A. Caoili E. M. (2014). Adrenocortical carcinoma. Endocr. Rev. 35 (2), 282–326. 10.1210/er.2013-1029 24423978 PMC3963263

[B13] Fassnacht M. Johanssen S. Quinkler M. Bucsky P. Willenberg H. S. Beuschlein F. (2009). Limited prognostic value of the 2004 international union against cancer staging classification for adrenocortical carcinoma: proposal for a revised TNM classification. Cancer 115 (2), 243–250. 10.1002/cncr.24030 19025987

[B14] Fay A. P. Elfiky A. Teló G. H. McKay R. R. Kaymakcalan M. Nguyen P. L. (2014). Adrenocortical carcinoma: the management of metastatic disease. Crit. Rev. Oncol. Hematol. 92 (2), 123–132. 10.1016/j.critrevonc.2014.05.009 24958272 PMC4578298

[B15] Gillison M. L. Akagi K. Xiao W. Jiang B. Pickard R. K. L. Li J. (2019). Human papillomavirus and the landscape of secondary genetic alterations in oral cancers. Genome Res. 29 (1), 1–17. 10.1101/gr.241141.118 PMC6314162 30563911

[B16] He Z. (2021). Bioinformatic analysis identifies prognostic genes associated with cancer stem cell properties in adrenocortical carcinoma. Zeng Guohua, editor-in-chief 2022, 1–67. 10.27043/d.cnki.ggzyc.2021.000317

[B17] Lam K. Y. (1992). Adrenal tumours in Chinese. Virchows Arch. A Pathol. Anat. Histopathol. 421 (1), 13–16. 10.1007/BF01607133 1636245

[B18] Lee J. Kim K. Ryu T. Y. Jung C. R. Lee M. S. Lim J. H. (2021). EHMT1 knockdown induces apoptosis and cell cycle arrest in lung cancer cells by increasing CDKN1A expression. Mol. Oncol. 15 (11), 2989–3002. 10.1002/1878-0261.13050 34214254 PMC8564652

[B4] Li H. Ji Z. Zhang B. Zhang Y. Lu L. Deng J. (2021). Expert consensus on diagnosis and treatment of adrenocortical carcinoma. Mod. J. urology 26 (11), 902–908. 10.3969/j.issn.1009-8291.2021.11.002

[B19] Li T. Yang J. Yang B. Zhao G. Lin H. Liu Q. (2020). Ketamine inhibits ovarian cancer cell growth by regulating the lncRNA-PVT1/EZH2/p57 Axis. Front. Genet. 11, 597467. 10.3389/fgene.2020.597467 33763107 PMC7982774

[B20] Li Z. Liu F. Y. Kirkwood K. L. (2020). The p38/MKP-1 signaling axis in oral cancer: Impact of tumor-associated macrophages. Oral Oncol. 103, 104591. 10.1016/j.oraloncology.2020.104591 32058294 PMC7136140

[B21] Libé R. (2015). Adrenocortical carcinoma (ACC): diagnosis, prognosis, and treatment. Front. Cell Dev. Biol. 3, 45. 10.3389/fcell.2015.00045 26191527 PMC4490795

[B22] Lu J. Xu J. Li J. Pan T. Bai J. Wang L. (2018). FACER: comprehensive molecular and functional characterization of epigenetic chromatin regulators. Nucleic Acids Res. 46 (19), 10019–10033. 10.1093/nar/gky679 30102398 PMC6212842

[B23] Marazzi I. Greenbaum B. D. Low D. Guccione E. (2018). Chromatin dependencies in cancer and inflammation. Nat. Rev. Mol. Cell Biol. 19 (4), 245–261. 10.1038/nrm.2017.113 29184195

[B24] Plass C. Pfister S. M. Lindroth A. M. Bogatyrova O. Claus R. Lichter P. (2013). Mutations in regulators of the epigenome and their connections to global chromatin patterns in cancer. Nat. Rev. Genet. 14 (11), 765–780. 10.1038/nrg3554 24105274

[B25] Qi E. Zhou Y. Dai Y. (2021). Using bioinformatics methods to explore key genes in adrenocortical carcinoma. J. Chongqing Med. Univ. 46 (04), 444–449. 10.13406/j.cnki.cyxb.002458

[B26] Shan Z. (2021). Bioinformatics methods for analysis of key gene expression and identification of osteosarcoma. Li Fuguang, Editor-in-chief. 2022, 1–65. 10.27466/d.cnki.gzzdu.2021.003470

[B27] Tang Y. Wang S. (2019). Research progress of AURKB in bladder cancer. J. Qiqihar Med. Coll. 40 (12), 1521–1524. 10.3969/j.issn.1002-1256.2019.12.031

[B28] Tang Y. Zhang X. Liu X. Mei D. Fu Q. Li J. (2020). Pharmacological effects and safety of mitotane in the treatment of adrenocortical carcinoma. J. Clin. Pharmacother. 18 (10), 68–71. 10.3969/j.issn.1672-3384.2020.10.015

[B29] Vaidia A. Nehs M. Kilbridge K. (2019). Treatment of adrenocortical carcinoma. Surg. Pathol. Clin. 12 (4), 997–1006. 10.1016/j.path.2019.08.010 31672303

[B30] Wang Z. Hausmann S. Lyu R. Li T. M. Lofgren S. M. Flores N. M. (2020). SETD5-Coordinated chromatin reprogramming regulates adaptive resistance to targeted pancreatic cancer therapy. Cancer Cell 37 (6), 834–849. e13. 10.1016/j.ccell.2020.04.014 32442403 PMC8187079

[B31] Watson Z. L. Yamamoto T. M. McMellen A. Kim H. Hughes C. J. Wheeler L. J. (2019). Histone methyltransferases EHMT1 and EHMT2 (GLP/G9A) maintain PARP inhibitor resistance in high-grade serous ovarian carcinoma. Clin. Epigenetics 11 (1), 165. 10.1186/s13148-019-0758-2 31775874 PMC6882350

[B32] Yang Y. Xi Z. Zhang L. (2022). Expression of Aurora kinase B and tumor protein 53 in hepatocellular carcinoma and their relationship with clinicopathological features and prognosis. Liver 27 (02), 188–192. 10.14000/j.cnki.issn.1008-1704.2022.02.006

[B33] Zennaro M. C. Jeunemaitre X. Boulkroun S. (2012). Integrating genetics and genomics in primary aldosteronism. Hypertension 60, 580–588. 10.1161/HYPERTENSIONAHA.111.188250 22802222

[B34] Zhang L. Lu J. Song W. Liu X. Liu F. Wang X. (2021). Screening of breast cancer-related prognostic genes based on TCGA database and its clinical significance. J. Qiqihar Med. Coll. 42 (21), 1841–1845. 10.3969/j.issn.1002-1256.2021.21.001

[B35] Zhou N. Fan R. Zhang S. Gu C. Lu W. (2022). Screening and prognostic analysis of key genes in adrenocortical adenocarcinoma based on GEO database. Mod. J. urology 27 (02), 161–165. 10.3969/j.issn.1009-8291.2022.02.016

