# Supplementary figures and images for: Identification of a chromatin regulator signature and potential prognostic ability for adrenocortical carcinoma
Source: Front Genet. 2022 Aug 26;13:948353. doi: 10.3389/fgene.2022.948353 (PMC9459121; doi:10.3389/fgene.2022.948353)

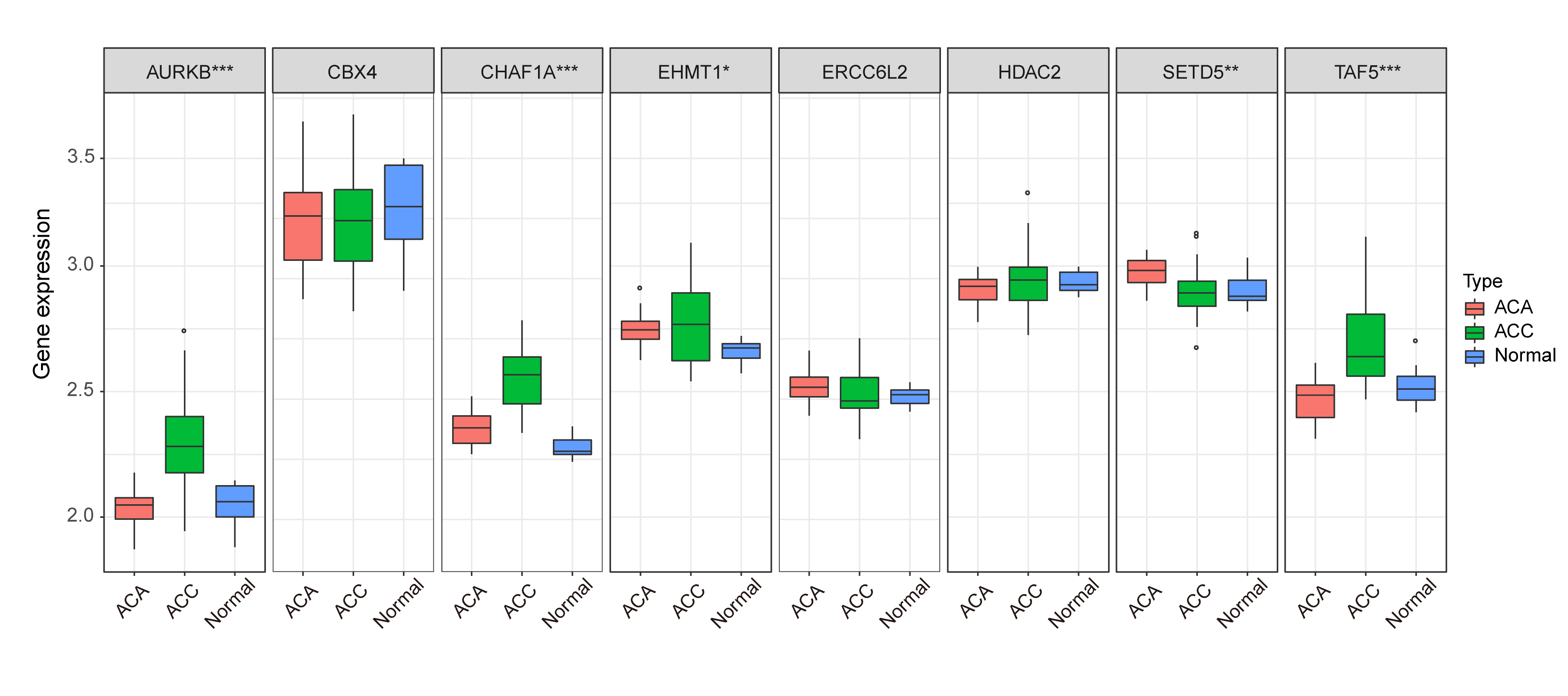

Supplement: Supplementary file 1 [file Image1.TIF]
